# Antiangiogenic drugs in ovarian cancer

**DOI:** 10.1038/sj.bjc.6604767

**Published:** 2008-11-11

**Authors:** G C Kumaran, G C Jayson, A R Clamp

**Affiliations:** 1 Department of Medical Oncology, Cancer Research UK and University of Manchester, Christie Hospital NHS Trust, Wilmslow Road, Manchester, UK

**Keywords:** angiogenesis, vascular endothelial growth factor, angiogenesis inhibitors, ovarian neoplasms, biomarkers, adverse effects

## Abstract

Ovarian cancer continues to be a major cause of morbidity and mortality in women. Antiangiogenic treatments have emerged as a promising strategy to treat ovarian cancer. This article reviews the rationale supporting the use of antiangiogenic treatments in ovarian cancer, the clinical development of this group of drugs and the toxicities specific to this modality of treatment.

Ovarian cancer is the fourth most common cause of cancer death in women. Worldwide, there are more than 190 000 new cases of ovarian cancer each year, accounting for around 4% of all cancers diagnosed in women. Incidence rates vary considerably, with the highest rates in the United States and Northern Europe and the lowest rates in Africa and Asia.

The majority of patients with ovarian cancer present late with advanced disease (FIGO stage III/IV) and in this group of patients, despite multimodality treatment with surgical debulking followed by platinum–taxane combination chemotherapy, median survival is only 3 years.

New treatment approaches are therefore urgently required to improve outcome in this disease and one promising strategy to have emerged has been the study of angiogenesis in ovarian cancer and the role of modulators of angiogenesis in its treatment.

Angiogenesis is the process of new blood vessel development and is crucial for the growth of tumours beyond 100–200 *μ*m in diameter as diffusion of nutrients and oxygen from nearby capillaries is inadequate beyond this point to sustain cell function (Folkman *et al*, 1989). Early in tumorogenesis, an ‘angiogenic switch’ is flipped whereby the previously closely maintained physiological balance that keeps the adult vasculature in a generally quiescent state is tipped towards angiogenesis with upregulation of pro-angiogenic growth factors such as VEGF (vascular endothelial growth factor), FGF-2 (fibroblast growth factor-2) and their receptors and downregulation of antiangiogenic factors like thrombospondin-1 and angiostatin (Hanahan and Folkman, 1996). Although sprouting angiogenesis is clearly the most important mechanism for tumour vascularisation, several other pathways have been identified including vessel co-option, vasculogenic mimicry and intussusceptive angiogenesis (for further information see Hillen and Griffioen, 2007).

The VEGF family of growth factors and its receptors constitute the most important signalling pathways in tumour angiogenesis and have been well characterised by research over the last two decades. Vascular endothelial growth factor-A was initially identified as a vascular permeability factor in 1983 and later characterised as an endothelial-specific mitogen by Ferrara and Henzel (1989). Subsequently, seven family members have been identified – VEGF-A to -E and placental growth factor (PlGF)-1 and -2. They signal through three tyrosine kinase receptors, VEGFR-1 to –3, to which the growth factors bind, leading to the dimerisation and activation of downstream signalling cascades. Both VEGFR-1 and -2 can promote angiogenesis and VEGFR-3 stimulation leads to lymphangiogenesis. Although VEGFR-1 has a 10-fold higher binding affinity for VEGF-A, its activation has less of an impact on the activation of intracellular signalling intermediates than VEGFR-2 (Waltenberger *et al*, 1994). A degree of specificity has been shown for growth factor-receptor binding, VEGF-B and PlGF-1 and -2 bind to VEGFR-1, whereas VEGF-A interacts with both VEGFR-1 and -2. Vascular endothelial growth factor-C and -D specifically bind to VEGFR-3. There is a general consensus, however, that VEGFR-2 is the dominant receptor in mediating the pro-angiogenic functions of VEGF-A and this pathway has been prioritised for the development of antiangiogenic therapies.

## Angiogenesis in ovarian physiology

Each female reproductive cycle involves rapid increases in ovarian and endometrial tissue mass and vascularity with subsequent regression in non-fertile cycles. These changes are promoted by co-ordinated interactions between steroid hormones and angiogenic factors. At the start of an ovulatory cycle, a number of ovarian follicles start the step by step process of maturation with the subsequent selection of one or two dominant follicles, which are characteristically of higher vascularity (Ramakrishnan *et al*, 2005). These dominant follicles subsequently release ova and afterwards turn into a temporary endocrine tissue (corpus luteum) synthesising the steroid hormones required for endometrial development. Angiogenesis is the key to follicular development and the degree of vascularity continues to increase during the luteal phase to supply nutrients and steroid precursors and allow the export of active steroid hormones to the endometrium. New vessel formation and development is therefore critical for the whole reproductive cycle, and the role of vascular growth factors involved in the regulation of this process is shown by their cyclical change corresponding to the various stages of the menstrual cycle. Intrafollicular levels of the VEGF family of proteins, in particular VEGF-A, increase during the initial part of the ovulatory cycle, with peak concentrations detected just before the start of the luteal phase. Treatment with VEGF inhibitors, including VEGF Trap, a soluble decoy receptor composed of parts of the growth factor-binding domains of VEGFR-1 and -2 fused to the Fc region of human IgG1 during the follicular phase, suppresses ovulation (Fraser *et al*, 2005).

The subsequent regression of the vasculature during follicular atresia is triggered by increases in the levels of angiogenic inhibitors, in particular upregulation of Ang-2, towards the end of the luteal phase (Ramakrishnan *et al*, 2005).

## Angiogenesis in ovarian cancer

Although microvascular density, a commonly used surrogate marker for tumour angiogenic potential, has not been shown to correlate convincingly with prognosis in ovarian cancer (Sonmezer *et al*, 2004; Gadducci *et al*, 2006), both pre-clinical and clinical studies investigating the VEGF family and its downstream signalling pathways have indicated the key importance of angiogenesis in the pathophysiology of ovarian cancer.

In animal models of ovarian cancer, it has been shown that VEGF blockade inhibits ascites formation and slows tumour growth (Byrne *et al*, 2003). Several retrospective clinical studies in ovarian cancer have also demonstrated that intratumoral VEGF and VEGFR-2 expression and the carriage of VEGF gene polymorphisms associated with an increased VEGF excretion are independent poor prognostic factors (Shen *et al*, 2000; Goodheart *et al*, 2005; Hefler *et al*, 2007).

Neuropilin-1 and -2 are cell surface proteins that bind to the commonest isoform of VEGF-A (VEGF-165) and may act as co-receptors to enhance VEGF signalling through VEGFR-1. Neuropilin expression is increased in ovarian carcinomas (Ferrara and Kerbel, 2005; Osada *et al*, 2006).

The intratumoral vasculature tends to be structurally and functionally abnormal with tortuous, leaky, dilated and immature vessels with poor flow. The endothelial cells in these newly formed vessels are more dependent on VEGF as a mitogen for survival than in mature vasculature elsewhere in the body (Kamba *et al*, 2006). It has been suggested that VEGF inhibition remodels and ‘normalises’ the intratumoral vasculature, leading to a better delivery of oxygen and concomitant chemotherapy to the normally hypoxic and acidic tumour milieu (Jain, 2001).

The inhibition of angiogenesis was initially proposed as a therapeutic target that may avoid the development of drug resistance. The genetic instability of tumour cells promotes the emergence of drug-resistant clones, whereas traditionally, this was considered unlikely to occur in ‘host’ endothelial cells that were thought to possess a stable genome. However, it is clear from the clinical studies discussed below that resistance to antiangiogenic treatment strategies is common and recent evidences suggest that in addition to morphological differences, tumour endothelial cells have distinct gene expression profiles (Seaman *et al*, 2007) compared with normal endothelium and can also be cytogenetically abnormal (Hida *et al*, 2004).

It is also now clear that VEGF receptors can be expressed and functional on cancer cells, indicating that anti-VEGF treatment strategies may have direct antitumour effects (Wedam *et al*, 2006).

## Targeting the vegf pathway in ovarian cancer

Given the apparent key importance of the VEGF signalling pathway in normal ovarian physiology and in ovarian cancer, results of clinical trials with agents targeting this pathway have been eagerly anticipated.

Bevacizumab, a humanised monoclonal antibody directed against VEGF, was the first antiangiogenic agent to be licensed in the treatment of cancer and has improved survival when administered in combination with chemotherapy in a broad range of epithelial malignancies (Hurwitz *et al*, 2004; Sandler *et al*, 2006; Miller *et al*, 2007). Although response rates to bevacizumab alone are very low in other tumour types, it has shown exciting single-agent activity at a dose of 15 mg kg^−1^ every 3 weeks in two phase II trials in patients with recurrent ovarian cancer, the majority of whom had platinum-resistant disease (Burger *et al*, 2007; Cannistra *et al*, 2007). Response rates of 16 and 21% were recorded with median progression-free survival of 4.4 and 4.7 months, significantly higher than those seen in previous phase II studies in this patient population. It is worth noting that however, the tolerability of bevacizumab differed in the two studies as discussed below. Bevacizumab has also been combined safely with carboplatin–paclitaxel chemotherapy in the first-line management of ovarian cancer (Penson *et al*, 2006), and this combination is currently being compared with chemotherapy alone in two large phase III trials (GOG-218 and ICON7). ICON7 is a two-armed trial comparing carboplatin and paclitaxel (six cycles) against carboplatin+paclitaxel+bevacizumab (7.5 mg kg^−1^, every 3 weeks) for six cycles followed by 12 cycles of maintenance bevacizumab. GOG-218 is a three-arm placebo-controlled study – all patients receive carboplatin and paclitaxel for six cycles – in the first experimental arm, patients also receive concurrent and maintenance bevacizumab (15 mg kg^−1^, every 3 weeks) for up to 16 doses, whereas the second experimental arm receives only concurrent bevacizumab followed by placebo maintenance.

The soluble decoy receptor, aflibercept (VEGF Trap), a fusion protein containing the VEGF-binding domains of both VEGFR-1 and -2 linked through the Fc region of human IgG1, is also under investigation as a single agent in relapsed platinum-resistant ovarian cancer with a response rate of 8% and acceptable toxicity being reported at a pooled interim analysis of a phase II study of two different dosing regimens (Tew *et al*, 2007).

**Figure 1:**
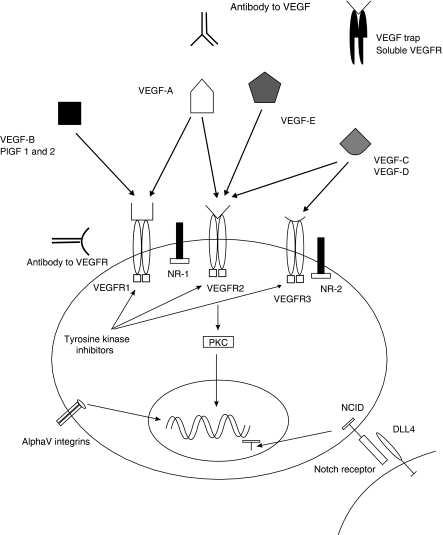
Strategies to inhibit the VEGF signalling pathway. Neuropilins (NRs) can function as co-receptors for vascular endothelial growth factor receptor (VEGFR). VEGF Trap (decoy receptor), growth factor-binding domains of VEGFR-1 and -2 bound to Fc fragment of IgG, and tyrosine kinase inhibitors (TKIs) prevent phosphorylation of VEGFR in response to VEGF binding. Delta-like ligand-4 (DLL4) binds to the Notch receptor, which leads to the cleavage of the Notch intracellular domain (NCID). The cleaved NCID translocates to the nucleus leading to the transcription of notch target genes. Protein kinase C (PKC) family of kinases are downstream mediators of VEGFR signalling. PlGF=placental-like growth factor.

Several small molecule tyrosine kinase inhibitors that target VEGFRs have now been investigated in the phase II setting in relapsed ovarian cancer and promising results have recently been presented (summarised in Table 1 and Figure 1). Intriguingly, the development of effusions was noted in some patients during the 2-week treatment break in sunitinib dosing probably due to the sudden release of VEGF inhibition, and so a continuous dosing regimen is now being explored (Biagi *et al*, 2008). Cediranib (AZD2171), which targets VEGFR-1, -2 and -3, is now being tested in platinum-sensitive relapsed ovarian cancer in a three-arm randomised placebo-controlled phase III trial in combination with carboplatin–paclitaxel (ICON6). Cediranib is administered concurrently with chemotherapy in both experimental arms, but is continued in a maintenance phase of up to 18 months duration in one of these (Table 1).

The use of combination anti-VEGF therapy has also been explored addressing the hypothesis that parallel inhibition at different points in the signalling pathway may translate into increased efficacy. A phase I study of sorafenib and bevacizumab demonstrated durable partial disease responses (4–22+ months) in 6 of 13 ovarian cancer patients recruited. Toxicity appears higher than that with single-agent anti-VEGF therapy, with two-thirds of the patients developing hypertension and 79% incidence of grade 1–3 hand–foot syndrome (Azad *et al*, 2008). Enteral fistulae were also seen in 2 of the 13 ovarian cancer patients on study.

## Toxicity with VEGF inhibitors

Treatments that interfere with VEGF function are generally well tolerated although a specific side-effect profile is associated with this treatment modality.

The most common toxicities are hypertension (grade 3 in approximately 10% patients), proteinuria (usually grade 1–2), haemorrhage, arterial and venous thrombotic events, impaired wound healing and, of particular concern in the initial phase II bevacizumab trials in ovarian cancer, gastrointestinal perforation.

Although the role of VEGF in normal endothelial homoeostasis is not well understood, the rapid regression of capillaries in several different tissues such as pancreatic islets, thyroid, adrenal cortex, choroid plexus and small intestinal villi is seen within a few days of starting treatment with VEGF inhibitors in animal models (Kamba *et al*, 2006). Loss of fenestration of renal glomerular capillaries also occurs, which might contribute to hypertension and proteinuria (Izzedine *et al*, 2007). Vascular endothelial growth factor also promotes endothelial nitric oxide production, and the removal of this stimulus may lead to vasoconstriction and hypertension (Baffert *et al*, 2006; Kamba *et al*, 2006).

Bowel perforation is a feared adverse event associated with bevacizumab and it was observed at a higher frequency in colorectal cancer patients receiving concurrent chemotherapy (1.5% cases compared with 0% of patients receiving chemotherapy alone) (Hurwitz *et al*, 2004). Patients with ovarian cancer are prone to bowel dysmotility and subacute bowel obstruction as a direct result of the pathophysiology of the disease. These characteristics may put ovarian cancer patients at a higher risk of having adverse events like perforation with VEGF inhibition. The data from the key phase II studies suggest that the risk of perforation is higher for patients who were heavily pre-treated with chemotherapy (more than three prior regimens). Although GOG-170D (Burger, 2007) did not report any cases of bowel perforation, eligible patients were limited to two prior chemotherapy regimens. The study of Cannistra *et al* (2007) was stopped early due to a perforation rate of 11.4% (5 of 44 patients). All perforations occurred in patients who had received three or more chemotherapy regimens and there was a trend towards higher perforation rates in patients with documented bowel wall involvement or bowel obstruction at study entry. Bowel perforations and enteral fistulae have also been reported in other phase II trials utilising bevacizumab in advanced ovarian cancer (Azad *et al*, 2008; Garcia *et al*, 2008). However, no definitive conclusions on aetiological factors can be drawn, due to the small number of patients in each group. Data from GOG-218 and ICON7 are awaited to clarify the risks of major gastrointestinal complications in the first-line setting.

Reversible posterior leukoencephalopathy syndrome and tracheo-oesophageal fistulae are significant, but rare, complications of bevacizumab treatment. Vascular endothelial growth factor receptor tyrosine kinase inhibitors have also been associated with clinical hypothyroidism, which could be caused by the inhibition of iodine uptake in the thyroid (Mannavola *et al*, 2007).

## Metronomic chemotherapy and VEGF inhibitors

Tumour endothelium, by virtue of its higher proliferation rate compared with the normal adult vasculature, makes itself a target for anticancer treatment. Cytotoxic chemotherapy is conventionally administered at a dose close to its maximum-tolerated dose, with breaks to allow the recovery of normal tissues. Chemotherapy at this dose does kill proliferating vascular endothelial cells, but recovery happens rapidly. In animal models, lower doses of chemotherapy for example, paclitaxel, vinorelbine and cyclophosphamide given frequently (metronomic dosing) target proliferating endothelial cells leading to their apoptosis with few side effects and no significant direct tumour cytotoxicity (Browder *et al*, 2000; Hanahan *et al*, 2000; Klement *et al*, 2000). In ovarian cancer, metronomic docetaxel chemotherapy in combination with AEE788, a combined EGFR and VEGFR inhibitor, has shown encouraging activity in an orthotopic mouse model utilising a cell line resistant to conventional chemotherapy dosing (Kamat *et al*, 2007). This approach has also been explored in a phase II clinical trial of bevacizumab (10 mg kg^−1^, every 2 weeks) and metronomic oral cyclophosphamide (50 mg daily) in platinum-resistant/partially platinum-sensitive ovarian cancer with promising activity. Median time to progression was 7.2 months and median survival time was 16.9 months in this study involving 70 patients (Garcia *et al*, 2008).

## Targeting angiogenesis through alternative pathways

Although strategies directly inhibiting VEGF have shown clinical activity and have confirmed angiogenesis inhibition as a therapeutic anticancer option, not all cancers respond to this approach and resistance inevitably develops in the clinical setting presumably through the upregulation of alternative pro-angiogenic pathways. Casanovas *et al* (2005) showed in an animal pancreatic islet cell tumour model that evasion of VEGFR-2 signalling blockade was achieved through the upregulation of FGF family members. Interestingly, preliminary data from a phase II trial of cediranib in glioblastoma indicated that circulating FGF-2 concentrations increased during drug holidays and on disease progression, reinforcing the potential importance of this resistance pathway (Batchelor *et al*, 2007). Several other resistance mechanisms have also been proposed including the recruitment of vascular progenitor cells and monocytes from bone marrow as well as the increased protection from better pericyte coverage (Bergers and Hanahan, 2008). Recent animal evidences also suggest that VEGFR-3 may have a role in driving angiogenic sprouting in the presence of VEGFR-2 inhibitors, suggesting a role for VEGF-C in mediating resistance to VEGFR-2 inhibition (Tammela *et al*, 2008). The development of alternative/combination antiangiogenic approaches is therefore vitally important and some of the more promising alternative targets are discussed below (see Figure 1).

The epidermal growth factor receptor (EGFR) is overexpressed in ovarian carcinoma, and EGFR signalling has been shown to upregulate VEGF expression. Although erlotinib (EGFR tyrosine kinase inhibitor) does not have any relevant single-agent activity in ovarian cancer, combined VEGF and EGFR inhibition is currently under investigation. The combination of erlotinib and bevacizumab has already been explored in a phase II study (Friberg *et al*, 2006), with two responses seen in 13 patients.

The platelet-derived growth factor (PDGF) signalling pathway is another potential target for antiangiogenic therapy, as PDGFR signalling is of key importance in maintaining the homoeostasis of pericytes, mesenchymally derived cells that surround capillaries and venules, and are vital for microvessel stability and function. Platelet-derived growth factor and PDGFR have also been identified in metastatic ovarian carcinoma, with overexpression of PDGFR associated with poor prognosis (Dabrow *et al*, 1998). Pre-clinical studies in ovarian cancer xenograft models have shown increased efficacy for the combination of VEGFR and PDGFR tyrosine kinase inhibitors associated with decreased vessel coverage by pericytes supporting PDGF signalling as an antiangiogenic therapeutic target (Lu *et al*, 2007). However, ‘pure’ PDGFR inhibition needs to be approached with caution as a phase II study of CDP860, a pegylated di-Fab’ molecule that binds to and blocks the activity of the *β*-subunit of PDGFR, was stopped early because of significant toxicity due to fluid accumulation (Jayson *et al*, 2005). Imaging studies demonstrated that CDP860 significantly increased the ratio of vascular volume to tumour volume, suggesting the recruitment of tumour-associated non-functioning vessels.

Another pathway that is of emerging importance in tumour angiogenesis is the Delta/Jagged-Notch system, which is involved in cell–cell interaction in multicellular organisms, and is required for the development of a normal cardiovascular system. The key receptor–ligand pair in tumour endothelium appears to be delta-like ligand-4 (DLL4) and Notch-1. Delta-like ligand-4 is normally induced by VEGF as a negative-feedback regulator of vascular growth and is strongly expressed in tumour vasculature. Delta-like ligand-4 blockade in animal xenograft models interestingly results in increased tumour vasculature but reduced tumour growth (Thurston *et al*, 2007). The vessels formed in this way have increased sprouting, with lack of maturation, and are too disorganised to accommodate adequate perfusion. Further studies need to be performed to delineate in detail the effects of DLL4 blockade on the normal vasculature, but it potentially represents a very exciting therapeutic target.

Vascular endothelial growth factor gene expression is influenced by several factors although hypoxia is vitally important. This leads to the activation of hypoxia-inducible factor-1*α*, which activates the transcription of multiple target genes including VEGF. Hypoxia-inducible factor-1*α* levels are in turn controlled by mammalian target of rapamycin (mTOR). Temsirolimus is an inhibitor of mTOR and is currently under investigation in a phase II study in recurrent ovarian cancer (GOG-0170I).

One of the key downstream mediators of VEGF signalling is the protein kinase C (PKC) family of serine/threonine kinases. Enzastaurin (LY317615) is an inhibitor of PKC-*β* that has shown activity in phase I studies with minimal toxicity and is being studied further in ovarian cancer (Carducci *et al*, 2006).

Volociximab (M200) is a monoclonal antibody that specifically binds *α*5*β*1 integrin, which is part of a family of transmembrane proteins that play a critical role in vasculogenesis (*α*5 integrins) (Takada *et al*, 2007). Pre-clinical testing showed inhibition of proliferating endothelial cells by volociximab and it is currently under phase II investigation as a single agent in platinum-resistant ovarian cancer.

## Future prospects and challenges in antiangiogenic treatment

The pre-clinical and clinical studies show promise for the antiangiogenic approach in ovarian cancer treatment. Many unanswered questions, however, remain as to the best strategy to use with these treatments. For instance, is combination with chemotherapy the best approach and, if so, is concurrent antiangiogenic therapy sufficient or should maintenance treatment be pursued? Hopefully, answers to these questions will emerge for bevacizumab from the ongoing phase III studies (ICON7 and GOG-218). Given the plethora of antiangiogenic targets and drugs under development, future research will also need to concentrate on the best approaches to combine/sequence these agents.

Currently, one of the biggest challenges in the clinical development of antiangiogenic agents is developing robust biomarkers for clinical use that will allow us to both ascertain the optimum biological dose of these agents in early clinical trials and also to select those patients who are most likely to benefit from antiangiogenic treatment. This is particularly important given the costs and not the insignificant toxicity profiles of these agents.

Dynamic contrast-enhanced magnetic resonance imaging (DCE-MRI) has been the most utilised pharmacodynamic imaging modality in early phase clinical trials of angiogenic inhibitors. This functional imaging technique is non-invasive and can be used to serially assess tumour vasculature *in vivo* (O'Connor *et al*, 2007). Although DCE-MRI evidence of drug-induced changes in the intratumoral vasculature has guided dose selection in the phase I setting, there is currently relatively little evidence that it can be used to predict clinical benefit.

[^18^F]fluorothymidine (FLT) PET imaging may be an early metabolic predictor of response to antiangiogenic therapy. In a phase II study of bevacizumab and irinotecan in recurrent glioblastoma, patients whose tumours demonstrated a metabolic response lived three times longer than non-responders (Chen *et al*, 2007), indicating that FLT-PET should be explored further.

Serological biomarkers for antiangiogenic treatment potentially have greater clinical utility, given their lower cost and the ease of repeated sampling compared with imaging strategies. Although changes in the levels of circulating pro-angiogenic growth factors in response to antiangiogenic drug exposure have been seen in several early phase clinical trials and a pharmacodynamic signal consistent with the inhibition of VEGFR-2 signalling (elevations in VEGF-A and PlGF associated with falls in soluble VEGFR-2 and -3) seems to be emerging from trials of VEGFR tyrosine kinase inhibitors (Batchelor *et al*, 2007; Rini *et al*, 2008), no clear pattern predictive of response or the subsequent development of treatment resistance has emerged yet. The serial assessment of a panel of circulating biomarkers is an integral part of the ICON7 trial, with the aim of identifying markers of sensitivity to both antiangiogenic therapy and early disease progression.

Circulating endothelial cell (CEC) and endothelial progenitor cell (CEP) concentrations have also been shown to be promising surrogate markers in pre-clinical studies (Shaked *et al*, 2005). In the clinic, the benefit from metronomic chemotherapy in metastatic breast cancer was associated with an elevation of CEC levels secondary to apoptotic cells 2 months after the commencement of the treatment (Mancuso *et al*, 2006). A subsequent study by the same group has suggested that an elevated CEC level before treatment commencement predicted clinical benefit from the combination of metronomic chemotherapy and bevacizumab in metastatic breast cancer (Dellapasqua *et al*, 2008). However, although these results are intriguing, it should be noted that the appropriate enumeration of CECs is technically challenging (Strijbos *et al*, 2008). Interestingly, comparative data from four phase II clinical trials of anti-VEGF agents presented at ASCO this year in which an identical technology was used to measure CECs indicated that changes in CEC/CEP levels did not demonstrate a consistent pattern but were dependent on the antiangiogenic agent used and the treatment context (Duda *et al*, 2008). Further studies to identify clinically useful predictive and pharmacodynamic biomarkers for angiogenesis and antiangiogenic therapy are urgently needed.

## Conclusions

Anti-VEGF drugs, in particular bevacizumab, have shown promising activity as single agents in chemotherapy-resistant ovarian cancer, supporting the strong pre-clinical rationale for the utility of this treatment strategy. Combinations with chemotherapy are currently being tested in clinical trials in the first-line setting and it is hoped that this approach may significantly improve patient outcome. Biomarkers of response, however, need to be studied to enable a better selection of patients who will benefit from treatment and to ascertain optimal dosing.

As more targets for antiangiogenic therapies emerge from pre-clinical studies, the interactions between these different pro-angiogenic pathways, the concept of combination antiangiogenic treatments and the evaluation of mechanisms of ‘resistance’ to angiogenesis inhibitors will need to be addressed in the future.

## Figures and Tables

**Table 1:** Phase II trials of oral VEGFR tyrosine kinase inhibitors in relapsed ovarian cancer

	**Eligibility criteria**	**Dose regimen**	**No. of patients**	**% Platinum resistant**	**Efficacy**	**Reported Gd3/4 toxicities**
Cediranib (Matulonis *et al*, 2008)	Up to two prior lines of therapy, ECOG PS 0/1	45 mg o.d. reduced to 30 mg o.d. (toxicity)	29	55	18.5% (response rate)	Hypertension (45%), fatigue (17%), diarrhoea (10%)
Cediranib (Hirte *et al*, 2008)	One prior line of therapy, ECOG PS 0–2	45 mg o.d. reduced to 30 mg o.d. (toxicity)	60	57	41% platinum sensitive, 29% resistant (response and disease stabilisation)	Hypertension (33%), fatigue (20%)
Sunitinib (Biagi *et al*, 2008)	Up to two prior lines of therapy	50 mg o.d. 4 out of 6 weeks	17	?	12% (PR), 59% (response/ stabilisation)	Fatigue, hand–foot syndrome, neutropaenia, thrombocytopaenia
Sorafenib (Matei *et al*, 2008)	Up to two prior lines of therapy, GOG PS 0–2	400 mg o.d.	73 (59 evaluable for response)	?	3% (PR), 20% (stable disease>6months)	Rash (17%), metabolic (15%), gastrointestinal (4%)
Pazopanib (Friedlander *et al*, 2007)	Relapsed disease after complete CA-125 response to first-line therapy, ECOG PS 0–1	800 mg o.d.	17	26	47% (CA-125 response)	Diarrhoea (12%), ALT elevation (12%)

ALT=alanine transaminase; ECOG=Eastern Cooperative Oncology Group; GOG=Gynecologic Oncology Group; o.d.=once a day; PR=partial response; PS=performance status.
